# Comparative Evaluation of Buccal Cortical Bone Thickness in the Posterior Mandible Across Skeletal Patterns: A CBCT Study in a Sample of Iranians

**DOI:** 10.1155/ijod/1205123

**Published:** 2026-04-30

**Authors:** Paniz Ranji, Kazem Dalaei, Melika Mansouri, Yaser Safi, Maede Jafarian Amiri, Mehdi Hosseinzadeh

**Affiliations:** ^1^ Department of Oral and Maxillofacial Radiology, School of Dentistry, Shahid Beheshti University of Medical Sciences, Tehran, Iran, sbmu.ac.ir; ^2^ Department of Orthodontics, School of Dentistry, Shahid Beheshti University of Medical Sciences, Tehran, Iran, sbmu.ac.ir; ^3^ School of Dentistry, Shahid Beheshti University of Medical Sciences, Tehran, Iran, sbmu.ac.ir; ^4^ Department of Oral and Maxillofacial Radiology, School of Dentistry, Ardabil University of Medical Sciences, Ardabil, Iran, arums.ac.ir

**Keywords:** cone-beam computed tomography, cortical bone thickness, miniscrew, orthodontic anchorage

## Abstract

**Purpose:**

This study aimed to evaluate buccal cortical bone thickness in the posterior mandible across different sagittal and vertical skeletal patterns using cone‐beam computed tomography (CBCT).

**Materials and Methods:**

In this retrospective observational study, cortical buccal bone thickness between posterior teeth was assessed at 4 and 7 mm distances from the alveolar crest using CBCT images of 112 Iranian participants aged 18–60 years. Participants were classified into six groups: Class I normal angle, Class I low angle, Class I high angle, Class II normal angle, Class II low angle, and Class II high angle. Statistical analyses included the Shapiro–Wilk test, Levene’s test, multivariate analysis of variance (MANOVA), paired‐sample *t*‐test, and intraclass correlation coefficient (ICC), with a significance level set at *α* = 0.05.

**Results:**

Cortical bone thickness generally increased from anterior to posterior regions and from the alveolar crest toward the basal jaw in Class II and both high‐ and low‐angle groups, with exceptions observed in Class I and normal‐angle individuals. Statistically significant differences in cortical bone thickness were observed between Class I and Class II skeletal patterns at specific posterior sites, with Class I individuals consistently exhibiting greater thickness. Differences related to vertical skeletal pattern were limited, observed only at the distal canine (4‐mm level). Sex, age, and side‐to‐side differences influenced cortical thickness in certain regions, with slightly greater thickness on the left side at some measurement sites.

**Conclusions:**

Buccal cortical bone thickness in the posterior mandible varies significantly with the sagittal skeletal pattern, whereas the vertical skeletal pattern has minimal influence.

## 1. Introduction

Anchorage in orthodontics is essential for preventing unwanted tooth movement and plays a critical role in the success of orthodontic treatment [1, 2]. Achieving maximum anchorage without displacement of the anchorage unit has long been a challenge in orthodontic biomechanics [3, 4]. To enhance anchorage control and facilitate efficient tooth movement, various devices such as palatal implants, miniplates, and miniscrews have been introduced [5].

Initially, miniscrews with diameters of ~2 mm were placed in different regions of the jaws to evaluate their stability. These devices offer several advantages, including small size, ease of insertion and removal, strong anchorage potential, low cost, and high patient acceptance. Nevertheless, miniscrew loosening during orthodontic treatment remains a concern, making primary stability a critical determinant of clinical success [6–8]. Miniscrew stability is influenced by multiple factors, including cortical bone thickness, bone density and depth, soft‐tissue quality and thickness, and proximity to adjacent anatomical structures [8, 9].

Skeletal factors—particularly the quality and quantity of cortical bone—play a pivotal role in miniscrew anchorage [10]. Unlike conventional dental implants, miniscrews rely predominantly on mechanical retention rather than osseointegration. Due to its greater strength and resistance to deformation, cortical bone provides more effective anchorage and contributes significantly to initial stability. Alveolar bone characteristics, especially buccal and lingual cortical bone thickness, are therefore critical determinants of miniscrew success. Previous studies have suggested that cortical bone thicknesses below 1 mm are associated with higher failure rates. Moreover, individuals with high‐ or normal‐angle mandibular patterns may exhibit thinner buccal cortical bone in the posterior mandible, increasing the risk of miniscrew failure. Consequently, identifying optimal insertion sites tailored to individual skeletal characteristics is essential [10–12].

Cone‐beam computed tomography (CBCT) has emerged as a valuable three‐dimensional imaging modality for evaluating bone morphology and thickness. CBCT enables accurate assessment of anatomical variations and precise localization of critical landmarks, such as the mandibular foramen, thereby reducing procedural risks and improving clinical outcomes [13, 14]. Additionally, a recent study used CBCT to evaluate both hard‐ and soft‐tissue thickness relevant to miniscrew insertion planning [15]. Its use in orthodontic diagnosis and treatment planning has increased substantially, with several studies confirming the high reliability and reproducibility of linear measurements obtained from CBCT images, demonstrating near‐perfect intra‐ and interexaminer agreement [16]. This imaging modality allows for precise evaluation of alveolar bone dimensions, which is essential for both implant planning and orthodontic anchorage applications. Recent CBCT investigations in Iranian samples have also highlighted skeletal pattern–related differences in mandibular morphology, such as condylar position and dimensions, further demonstrating the value of three‐dimensional assessment for orthodontic diagnosis and treatment planning [17].

CBCT‐based analyses have shown that cortical bone thickness varies across different jaw regions, with the posterior mandible generally exhibiting the greatest thickness, followed by the anterior mandible, anterior maxilla, and posterior maxilla. These regional variations highlight the importance of individualized treatment planning. Recent CBCT studies have underscored these differences in relation to facial skeletal patterns relevant to miniscrew placement [18]. By identifying optimal miniscrew placement sites, CBCT can help minimize the risk of anchorage failure and improve treatment predictability [19].

Cortical bone thickness in the buccal, lingual, or palatal regions may vary according to factors such as tooth position, root inclination, occlusal forces, skeletal pattern, and ethnicity. Previous studies have reported significant differences in cortical bone thickness among various skeletal patterns and populations [20]. However, findings in the literature remain inconsistent, and data focusing on the Iranian population are limited, despite known ethnic variations in craniofacial bone morphology. Establishing a clearer relationship between skeletal patterns and buccal cortical bone thickness may enhance miniscrew placement strategies and improve orthodontic treatment planning. Inadequate anchorage planning can lead to prolonged treatment duration, increased costs, and patient dissatisfaction, underscoring the clinical relevance of this investigation.

We hypothesized that cortical bone thickness in the posterior mandible differs among skeletal patterns, with Class I individuals exhibiting greater thickness than Class II individuals. This hypothesis is supported by previous evidence indicating that sagittal and vertical skeletal patterns influence craniofacial bone morphology. Understanding these variations is essential for optimizing miniscrew placement and improving orthodontic outcomes. Therefore, the aim of this study was to evaluate the relationship between buccal cortical bone thickness in the posterior mandible and sagittal and vertical skeletal patterns in an Iranian population using CBCT. By analyzing six distinct skeletal groups, this study seeks to contribute clinically relevant data to guide more precise and effective orthodontic anchorage planning.

## 2. Methods and Materials

### 2.1. Study Design and Ethical Approval

This study was designed as a retrospective observational investigation based on previously acquired CBCT images. A total of 112 CBCT scans were retrospectively selected from patients referred to the School of Dentistry, Shahid Beheshti University of Medical Sciences. All CBCT images had been obtained for routine diagnostic purposes and were subsequently analyzed for research objectives. The study protocol was approved by the Ethics Committee of Shahid Beheshti University of Dental Medicine (approval number: IR.SBMU.DRC.REC.1401.021). All CBCT datasets were anonymized to ensure patient confidentiality.

### 2.2. CBCT Acquisition Protocol

At the time of image acquisition, patients had been instructed to follow a standardized protocol, including remaining still, avoiding swallowing, and refraining from head movement during image acquisition. CBCT scans were obtained using a NewTom GiANO system (Verona, Italy) with the following parameters: 16 × 18 cm field of view (FOV), 90 kVp, 6 mA, and 15 s of exposure time. The estimated effective dose was within the range reported by the manufacturer for large‐FOV CBCT scans. Images were acquired with a voxel size of ~0.15 mm, providing sufficient spatial resolution for accurate identification of anatomical landmarks. All scans were reviewed for image quality by an oral and maxillofacial radiologist. All CBCT images were originally obtained for clinical diagnostic purposes, and only scans with a large FOV encompassing the mandible, maxilla, and facial structures were included in the analysis.

### 2.3. Study Population

**Figure 1:**
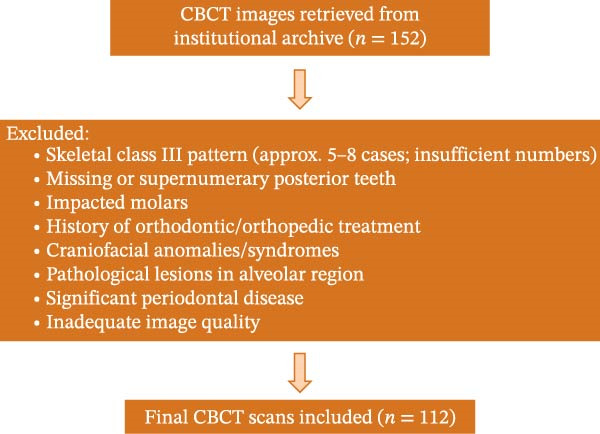
Flow diagram of CBCT sample selection.

CBCT images were retrospectively screened from the institutional imaging archive of the School of Dentistry, Shahid Beheshti University of Medical Sciences. Only scans that met the predefined inclusion criteria and demonstrated adequate image quality were included. A series of exclusion criteria were applied, including a history of orthodontic or orthopedic treatment; subjects with conditions associated with pronounced mandibular asymmetry, including craniofacial anomalies, facial trauma, and syndromic conditions (except mild or physiological mandibular asymmetry); oral habits; syndromes; history of trauma; cleft lip and palate; previous preprosthetic surgery in the posterior mandible; supernumerary or missing teeth; pathological lesions; dental crowding; impacted third molars; and clinically significant periodontal disease. Following the screening process, 112 CBCT scans from individuals aged 18–60 years with complete permanent dentition up to the second mandibular molar were included in the final analysis. These strict inclusion and exclusion criteria were applied to reduce potential selection bias. The screening and exclusion process, including the exclusion of skeletal Class III cases, is illustrated in a flow diagram consistent with STROBE recommendations (Figure 1). Age and sex distributions were evaluated across skeletal groups to ensure comparability (Table 1).

**Table 1:** Distribution of participants by skeletal group according to sex and mean age.

Skeletal group	Male	Female	Total number	Mean age
Class I normal angle	3	14	17	36.47
Class I low angle	7	13	20	30.85
Class I high angle	5	12	17	34.88
Class II normal angle	7	13	20	34.25
Class II low angle	8	11	19	33.57
Class II high angle	4	15	19	32.68

### 2.4. Skeletal Classification

The participants were classified into three vertical groups based on Jarabak’s ratio [(Posterior facial height × 100)/Anterior facial height] and the SN–MeGo angle (the angle between the SN and MeGo planes). Sagittal skeletal classification was assessed using the Wits appraisal rather than the ANB angle. In the Wits analysis, points A and B were projected perpendicularly onto the functional occlusal plane, and the linear distance between these projections was measured. In this study, Wits values between −1 and +1 mm were classified as skeletal Class I, while values greater than +1 mm were classified as skeletal Class II, in accordance with published criteria [21]. Based on the Wits values, participants were categorized into skeletal Class I or Class II. Consequently, six skeletal groups were defined: Class I normal angle, Class I low angle, Class I high angle, Class II normal angle, Class II low angle, and Class II high angle.

### 2.5. Cephalometric Criteria

• Jarabak’s ratio: values between 62% and 65% indicated a normal vertical pattern, values >65% indicated a short‐face pattern, and values <62% indicated a long‐face pattern.
• SN–MeGo angle: normal values ranged from 28.5° to 39.5°. Angles <28.5° indicated a short facial pattern, whereas angles >39.5° indicated a long facial pattern.
• Wits appraisal: values within the normal range were classified as skeletal Class I, while increased positive values were classified as skeletal Class II.

The Wits appraisal was selected due to its lower sensitivity to variations in cranial base length and nasion position, providing a more reliable sagittal skeletal assessment than angular measurements.

Cephalometric landmarks and measurements were reconstructed from lateral cephalometric images generated from the CBCT datasets using OnDemand3D software (Cybermed, Seoul, Korea). Image orientation and superimposition were verified using stable anatomical landmarks to ensure consistency between the right and left sides.

### 2.6. Measurement Procedure

CBCT datasets were reoriented in OnDemand3D software (Cybermed, Seoul, Korea) to standardize head position across subjects. The Frankfort horizontal plane (FH) was aligned parallel to the floor, the midsagittal plane passed through the nasion and anterior nasal spine, and the occlusal plane was leveled. Superimposition was verified using stable anatomical landmarks, including the mandibular condyles, mental foramen, and alveolar crest, to ensure consistent orientation between the right and left sides. All subsequent measurements of cortical bone thickness were performed relative to these standardized reference planes. Reconstructed panoramic CBCT images were used to identify interradicular spaces by drawing vertical reference lines parallel to the long axes of adjacent roots. Axial views were used to confirm that these lines bisected the interradicular space, and cross‐sectional views were employed to measure buccal cortical bone thickness. Buccal cortical bone thickness was measured as the shortest linear distance measured perpendicular to the outer buccal cortical surface, extending from the external cortical boundary to the point where cortical bone transitioned into trabecular bone. Measurements were performed after standardized image reorientation, ensuring that the measurement direction was consistently perpendicular to the local cortical surface, thereby minimizing the influence of cortical curvature. Measurements were performed between the roots of the canine and second molar, as well as at the distal aspect of the second molar. Vertical measurement levels were set at 4 and 7 mm from the alveolar crest (Figure 2). Measurements from the right and left sides were recorded and analyzed independently to account for potential side‐related anatomical variability. In cases where the third molar was absent, measurements were taken 1 mm distal to the root of the second molar.

Figure 2CBCT images: (A) reconstructed panoramic view with the target interdental region marked and a vertical reference line drawn; (B) axial section demonstrating that the vertical line bisects the interradicular space; and (C) cross‐sectional view. Measurement of cortical bone thickness in the buccal side of the mandible.

**Figure:**
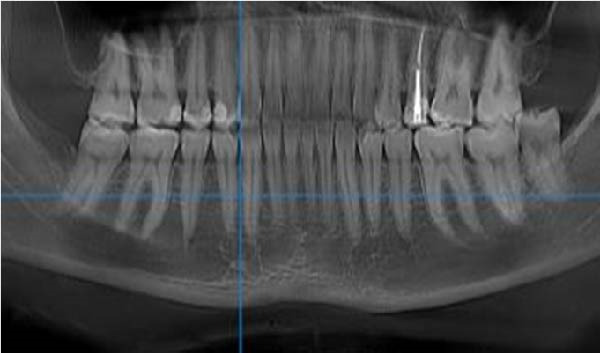
(A)

**Figure:**
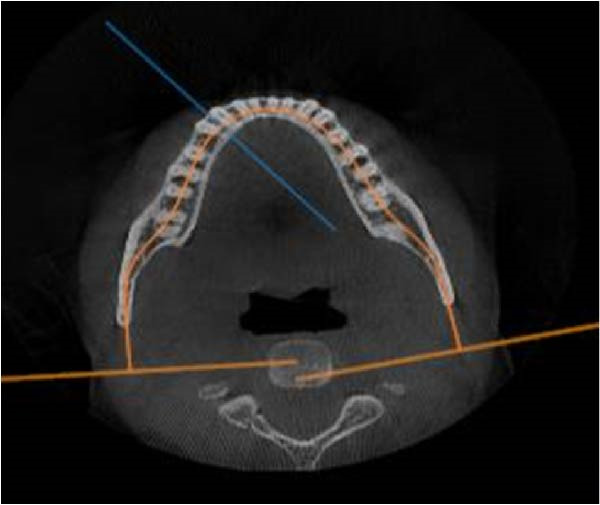
(B)

**Figure:**
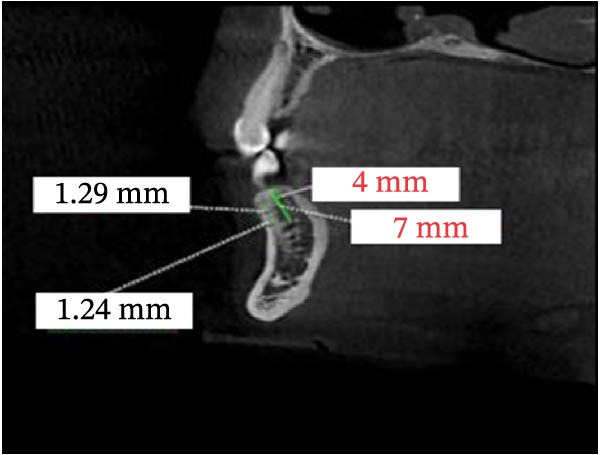
(C)

### 2.7. Reliability Assessment

All measurements were performed by a trained dental student under the supervision of experienced faculty members in oral and maxillofacial radiology and orthodontics. To assess intra‐examiner reliability, measurements were repeated after a 2‐week interval. For interexaminer reliability, 10 randomly selected CBCT scans were remeasured by a specialist in oral and maxillofacial radiology. Intraclass correlation coefficient (ICC) values were 0.91 for intra‐examiner reliability and 0.89 for interexaminer reliability, indicating excellent measurement reproducibility.

### 2.8. Sample Size and Data Collection

The study included six skeletal groups derived from two sagittal classes and three vertical patterns. Sample size estimation was performed using one‐way analysis of variance (ANOVA) in PASS 2021 software, assuming a Type I error rate of α = 0.05 and a Type II error rate of β = 0.20 (power = 80%). The estimated means and standard deviations for mandibular buccal cortical bone thickness were based on a previously published CBCT study with a similar adult population [12]. Based on estimated means and standard deviations, a minimum of 17 samples per group was required. To increase statistical robustness, a total of 112 CBCT scans were included.

### 2.9. Statistical Analysis

Data were extracted using OnDemand3D software and analyzed with SPSS software (version 26; IBM Corp., Armonk, NY, USA). The Shapiro–Wilk test assessed normality, and Levene’s test evaluated homogeneity of variances. Multivariate analysis of variance (MANOVA) was performed to assess the main and interaction effects of sagittal class (Class I vs. Class II) and vertical pattern (low, normal, and high) on the dependent variables. Age and sex were included as covariates in the general linear model. Post hoc analyses were conducted where appropriate. Paired‐sample *t*‐tests were specifically used to assess left–right differences in buccal cortical bone thickness. Statistical significance was set at α = 0.05. The ICC was used to evaluate measurement reliability.

## 3. Results

Out of 152 scans initially screened, a total of 112 CBCT scans were analyzed across six skeletal groups, comprising two sagittal classes and three vertical skeletal patterns (Table 1). Ten buccal cortical bone thickness variables were measured bilaterally for each participant, resulting in 20 measurements per subject.

An overall increasing trend in buccal cortical bone thickness was observed from anterior to posterior regions and from the alveolar crest toward the basal jaw in Class II individuals, as well as in low‐ and high‐angle vertical skeletal groups. In contrast, Class I individuals demonstrated an exception to this pattern: buccal cortical thickness at the distal aspect of the first premolar at 4 mm from the alveolar crest was greater than that measured at the distal second premolar and first molar at the same height and also exceeded the corresponding interradicular measurements at the 7 mm level. A similar pattern was observed in normal‐angle subjects. This localized increase reflects site‐specific variation in cortical bone morphology observed within the Class I skeletal pattern.

**Figure 3:**
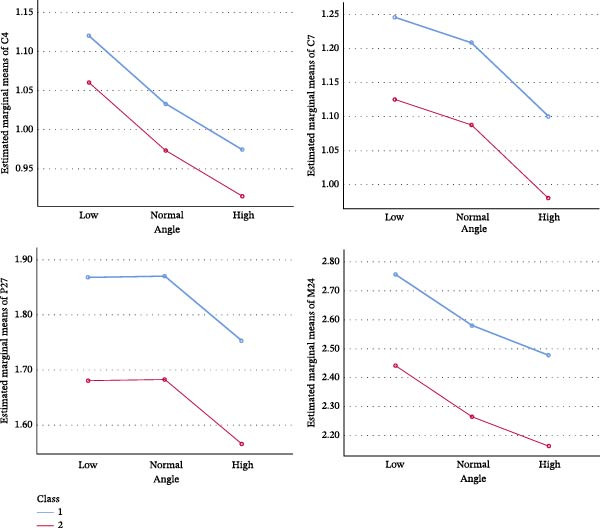
Measured thickness at the distal of the canine at 4 and 7 mm, second premolar at 7 mm, and second molar at 4 mm from the alveolar crest.

Multivariate analysis revealed that sagittal skeletal classification had a significant effect on buccal cortical bone thickness at selected posterior mandibular sites (C.7, P2.7, and M2.4), with Class I individuals exhibiting significantly greater thickness than Class II individuals. Vertical skeletal pattern showed a limited influence, reaching statistical significance only at C.4, where low‐angle individuals exhibited greater cortical thickness than high‐angle individuals (Figure 3). No significant interaction effects between sagittal and vertical skeletal classifications were detected. After adjustment for covariates, age and sex demonstrated site‐specific effects. Sex‐related differences were identified at the distal canine, first premolar, and first molar at the 7‐mm level; however, the predominance of female participants in some groups may limit the generalizability of sex‐specific findings. Age was significantly associated with cortical thickness at the distal canine (4 and 7 mm), first premolar (7 mm), and second molar (4 mm) (Table 2).

**Table 2:** MANOVA results showing the effects of sagittal and vertical classifications, sex, and age on buccal cortical bone thickness at different measurement sites.

Factor	Measurement site	F	*p*‐Value
Sagittal class	C.7	4.69	0.033
	P2.7	6.25	0.014
	M2.4	7.17	0.009

Vertical class	C.4	3.29	0.041

Gender	C.7	4.84	0.030
	P1.7	4.70	0.032
	M1.7	8.07	0.005

Age	C.4	10.16	0.002
	C.7	7.48	0.007
	P1.7	12.94	0.001
	M2.4	4.67	0.033

*Note:* C.4, 4 mm from alveolar crest on distal of canine. C.7, 7 mm from alveolar crest on distal of canine. P1.4, 4 mm from alveolar crest on distal of first premolar. P1.7, 7 mm from alveolar crest on distal of first premolar. P2.4, 4 mm from alveolar crest on distal of second premolar. P2.7, 7 mm from alveolar crest on distal of second premolar. M1.4, 4 mm from alveolar crest on distal of first molar. M1.7, 7 mm from alveolar crest on distal of first molar. M2.4, 4 mm from alveolar crest on distal of second molar. M2.7, 7 mm from alveolar crest on distal of second molar.

Paired comparisons between the right and left sides of the mandible revealed no significant differences in buccal cortical bone thickness at most measurement sites (*p*  > 0.05). However, statistically significant side‐related differences were observed at the distal aspects of the first and second molars at 7 mm from the alveolar crest, with greater cortical thickness on the left side (*p* = 0.004 and *p* = 0.048, respectively), indicating localized side‐related anatomical variability rather than generalized mandibular asymmetry.

## 4. Discussion

The use of mini‐implants for orthodontic anchorage has emerged as a relatively recent advancement, offering significant advantages and prompting ongoing investigation into methods to enhance their success. Several factors contribute to mini‐screw stability, including placement precision, implant design, mechanical retention, and the quality and quantity of cortical bone at the insertion site. These factors collectively influence both immediate stability and long‐term outcomes [22].

Previous studies have used various anatomical landmarks as reference points, such as the cemento‐enamel junction (CEJ) [23] and the alveolar crest [24]. The alveolar crest is the most commonly used reference, which is why it was selected in our study. To maintain consistency with prior research, bone thickness was assessed at 4 mm and 7 mm from the crest. These sites were also chosen due to the presence of attached gingiva, which reduces the risk of inflammation around mini‐implants [12, 20].

Our findings revealed significant differences in cortical bone thickness between Class I and Class II skeletal groups at key placement sites (7‐mm distal to the canine and 4‐mm distal to the second molar), with Class I individuals exhibiting greater thickness (*p*  < 0.05). This suggests that skeletal classification significantly influences cortical bone dimensions, impacting mini‐screw primary stability. In cases with insufficient thickness, clinicians may consider alternative strategies, such as angled placement (30°–60°), using narrower or longer screws, or selecting alternative sites like the buccal shelf or infrazygomatic crest.

Interestingly, vertical skeletal patterns showed limited effects, with significance only at C.4, where low‐angle individuals had thicker cortical bone than high‐angle individuals. This is consistent with the findings of Daoui et al. [18], who reported progressive changes in mandibular cortical thickness across vertical facial types without statistically significant differences. No significant interaction effects between sagittal and vertical classifications were detected. These findings align partially with previous studies. For example, Rossi et al. [25] reported no significant differences in buccal cortical thickness across sagittal groups, while Golshah et al. [26] observed significant sagittal differences, consistent with our results. This suggests that ethnic and population‐specific factors may influence cortical bone morphology. Similar patterns were noted in studies by Schneider et al. [24] and Hasani et al. [27], indicating limited vertical skeletal effects in the mandibular buccal region.

The use of CBCT provided reliable three‐dimensional assessments of cortical bone thickness, with measurements standardized relative to the Frankfort horizontal and midsagittal planes to ensure reproducibility. However, limitations exist, including the lack of clinical validation for converting grayscale values to Hounsfield units [28]. Recent CBCT evaluations have also highlighted optimal miniscrew insertion site characteristics in adult samples [29]. Additionally, due to insufficient Class III samples, this group was excluded. Future studies should include larger and more diverse samples, possibly incorporating anterior regions for comprehensive assessment.

Regarding the potential impact of infraeruption or supraeruption, all linear measurements were performed at fixed distances (4 and 7 mm) from the alveolar crest rather than the occlusal plane. This approach minimized variability due to tooth position changes associated with different vertical skeletal patterns, although subtle variations may still influence local alveolar morphology. Cortical thickness was measured perpendicular to the outer buccal cortical surface, minimizing effects of cortical curvature.

Several studies, including a meta‐analysis by Marquezan et al. [30], have identified a correlation between mini‐implant stability and cortical bone thickness. Both vertical and horizontal skeletal patterns independently contribute to cortical thickness variations [1, 2]. While prior studies often focused on either sagittal or vertical dimensions, our study examined the influence of both in a sample of Iranian individuals. Future research may explore combined effects of sagittal and vertical patterns on cortical thickness across posterior mandibular interradicular spaces using CBCT, alongside long‐term outcomes of mini‐screw success or failure.

While our study found no significant gender differences overall, sex and age can influence cortical bone morphology. Goyushov et al. [31] demonstrated that males had significantly greater cortical thickness than females (*p*  < 0.05), and younger adults (18–30 years) had lower mandibular bone density compared to older adults (*p*  < 0.01). This suggests that although skeletal classification (Class I vs. II) was the primary driver of differences in our study, sex and age may contribute to variability in bone quality.

Side‐to‐side comparisons revealed minor asymmetries, with greater cortical thickness observed on the left at the distal first and second molars (7 mm). This likely reflects natural anatomical variation rather than generalized mandibular asymmetry.

In summary, cortical bone thickness in the posterior mandible is significantly influenced by sagittal skeletal classification, with Class I individuals exhibiting greater thickness in key regions. Vertical skeletal patterns, age, and sex have localized effects. These findings have important clinical implications for mini‐screw placement. In Class II individuals with thinner cortical bone, alternative strategies such as angled insertion, narrower screws, or alternative sites may enhance primary stability. Future studies should examine combined effects of skeletal patterns and evaluate long‐term mini‐implant outcomes to refine clinical guidelines.

## 5. Limitations

This study has several limitations. The sample did not include sufficient Class III individuals, limiting generalizability to this skeletal group. Although CBCT provides reliable linear measurements, subtle variations in tooth eruption or alveolar morphology may still influence cortical bone assessment, particularly in extreme vertical patterns. Additionally, CBCT grayscale values cannot be directly converted to Hounsfield units, preventing quantitative bone density evaluation. Measurements were limited to specific interradicular sites in the posterior mandible, and findings may not apply to other regions, such as the anterior mandible or maxilla. The relatively wide age range (18–60 years) may influence cortical bone thickness and density. Although age was included as a covariate in the statistical analyses to adjust for its potential effect, residual age‐related variability may still exist. The predominance of female participants limits the generalizability of sex‐specific findings, and conclusions regarding sex differences should be interpreted cautiously. Finally, while age and sex were accounted for, other factors affecting bone quality, such as systemic health or long‐term periodontal history, were not controlled. Future studies with larger, more diverse samples and longitudinal follow‐up of mini‐screw stability are warranted.

## 6. Conclusion

This study highlights the significant relationship between buccal cortical bone thickness and skeletal patterns in the Iranian population. Class I individuals consistently exhibited greater cortical thickness than Class II subjects at key posterior mandibular sites. Sex and age demonstrated localized effects on bone thickness, whereas vertical skeletal pattern had minimal influence. These findings emphasize the importance of using CBCT for individualized orthodontic treatment planning, particularly in determining optimal mini‐screw placement. Such insights could help improve the success rate of anchorage devices and reduce treatment complications.

## Funding

No funding was received for this manuscript.

## Conflicts of Interest

The authors declare no conflicts of interest.

## Data Availability

The data that support the findings of this study are available from the corresponding author upon reasonable request.
